# *Notes from the Field*: Schedule I Substances Identified in Nootropic Gummies Containing *Amanita muscaria* or Other Mushrooms — Charlottesville, Virginia, 2023–2024

**DOI:** 10.15585/mmwr.mm7328a3

**Published:** 2024-07-18

**Authors:** Avery Michienzi, Jeremy Hamlin, Rita Farah, Lindsay Bazydlo

**Affiliations:** ^1^Department of Emergency Medicine, Division of Medical Toxicology, University of Virginia, Charlottesville, Virginia; ^2^University of Virginia Health Medical Laboratories, Charlottesville, Virginia; ^3^Department of Pathology, University of Virginia, Charlottesville, Virginia.

**Summary**

**What is already known about this topic?**

Gummies listing the hallucinogenic mushroom *Amanita muscaria* or other unnamed mushrooms as ingredients have been marketed as “nootropics” (substances taken to enhance cognitive function). *A. muscaria* can cause hallucinations, agitation, gastrointestinal upset, and seizures.

**What is added by this report?**

During September 2023–June 2024, five persons required hospital evaluation after ingesting gummies labeled to contain *A. muscaria*. Five brands of gummies marketed as mushroom-containing nootropics were analyzed; three contained unlabeled Drug Enforcement Administration schedule I substances psilocybin and psilocin.

**What are the implications for public health practice?**

Health care providers and the public should be aware that edible products marketed as mushroom-containing nootropics might contain undisclosed ingredients and have been linked to severe illness. Persons who experience symptoms after consuming these products should seek immediate medical attention.

Since late 2023, according to conversations between investigators and smoke shop employees, gas stations and smoke shops in Virginia have been selling mushroom gummies, marketed as nootropics (substances taken to enhance cognitive function) or psychedelics. These gummies are labeled to contain either *Amanita muscaria* or proprietary mushroom nootropic blends. Unlike other hallucinogenic mushrooms that contain the Drug Enforcement Administration (DEA) schedule I substance psilocybin, *A. muscaria* is currently legal.* *A. muscaria* contains ibotenic acid and muscimol and is used less commonly as a hallucinogen than are psilocybin-containing mushrooms because *A. muscaria* can cause undesired symptoms, including gastrointestinal upset, agitation, and seizures (*1**–**3*). During September 2023–June 2024, five patients, including one child, were evaluated in Virginia after ingestion of gummies listing *A. muscaria* as an ingredient. Because the actual contents of these novel mushroom gummies are unknown, samples from five brands were obtained for testing using liquid chromatography–mass spectrometry.

## Investigation and Outcomes

During September 1–November 20, 2023, the Blue Ridge Poison Center (BRPC) in Charlottesville, Virginia managed four cases of illness in adults who had intentionally ingested mushroom gummies labeled to contain the *A. muscaria* mushroom and were evaluated in an emergency department (ED). All patients experienced tachycardia, confusion, anxiety or somnolence, and nausea, and one patient reported chest pain. Two patients received benzodiazepines, three received antiemetics, and all four received intravenous fluids. All patients were discharged from the ED within 12 hours. In June 2024, BRPC managed an accidental ingestion of two *A. muscaria* mushroom gummies by a child aged 3 years who was hospitalized with symptoms of somnolence and vomiting. The child required no interventions and was discharged 1 day later.

All patients managed by BRPC were reported to ingest different mushroom gummy brands, but all brands taken by the patients were labeled to contain muscimol,^†^ ibotenic acid,^§^ and muscarine.^¶^ To determine whether these mushroom nootropics also contained other substances, investigators purchased six packages (five different brands) during October at gas stations and smoke shops near BRPC for testing. Because the specific brands reported by the patients were not available at these shops, investigators purchased three brands listing the same ingredients. In addition, two brands that were labeled to contain unspecified mushroom nootropics but were not labeled with *A. muscaria* were also purchased. Samples were analyzed by the University of Virginia Health Toxicology Laboratory using liquid chromatography–quadrupole time of flight on a Sciex X500R mass spectrometer. A qualitative untargeted approach was employed using independent data acquisition and substances identified using retention times, mass accuracy, and library matching.**

Mushroom gummies in four of the six bags tested were found to contain unlabeled psilocybin or psilocin, both of which are schedule I substances not currently legal in Virginia, where they were sold (Table). Additional unlabeled substances were found, including caffeine, ephedrine, and mitragynine (an opioid agonist commonly known as kratom). Ibotenic acid, muscimol, and muscarine were not present in the matching library, and their presence in the gummies was undetermined.

**TABLE:** Analysis* of contents of five brands of mushroom gummies marketed as nootropics and listing *Amanita muscaria *or other mushrooms as ingredients — Charlottesville, Virginia, 2023

Brand	Listed product ingredients	Identified compounds*
Diamond Shruumz Sour Peach Apple	Mushroom nootropic,^†^ caffeine, and lion’s mane, chagas, and reishi mushrooms	Psilocin
Diamond Shruumz Rainbow	Mushroom nootropic,^†^ caffeine, and lion’s mane, chagas, and reishi mushrooms	Psilocin and caffeine
Urb Magic Amanita Mushroom Watermelon	Muscimol, ibotenic acid, muscarine, and *A. muscaria* extracts	Psilocybin, psilocin, hordenine, and 2-phenethylamine
Wonderland Legal Psychedelics Cherry Nirvana	*A. muscaria* extract, blue lotus extract, and reishi, lion’s mane, and cordyceps mushrooms	Psilocin, N,N-dimethyltryptamine, caffeine, and mitragynine
Psilly’s Legal Psychedelic Mushrooms Fruit Punch	Fly agaric (*A. muscaria*) extract and hemp-derived extract	Ephedrine
Tryp mushroom gummies	Lion’s mane, reishi, cordyceps, maitake, and turkey tail mushroom extract and hemp extract	None

* Analysis conducted in the University of Virginia Health Toxicology Laboratory using liquid chromatography–quadrupole time of flight mass spectrometry. The testing instrument did not have capability to test for muscimol, ibotenic acid, muscarine, or the mushrooms indicated as ingredients on the product labels, although those mushrooms (lion’s mane, chagas, reishi, cordyceps, maitake, and turkey tail) are not psychogenic.

^†^ Unspecified mushroom ingredients used to enhance cognition.

## Preliminary Conclusions and Actions

The presence of the DEA schedule I substances psilocybin and psilocin in products legally sold at retail shops in Virginia represents a potential risk to the public. Further, the presence of mitragynine in one product is concerning because repeated mitragynine ingestion can increase the risk for opioid dependence (*4*). Whether persons who ingest gummies indicated to contain *A. muscaria* or other nootropics are aware that they might be receiving psilocybin or other substances represented as *A. muscaria* or if they desire the *A. muscaria* itself is unknown. Separate from the investigation described in this report, on June 12, 2024, CDC released a health advisory reporting that CDC, the Food and Drug Administration, and America’s Poison Centers^††^ are investigating cases of severe acute illnesses potentially associated with consuming one brand of mushroom gummies and chocolate bars, and are providing guidance for clinicians, public health practitioners, and the public.^§§^ Persons who believe they are purchasing gummies containing *A. muscaria* or other mushroom-containing gummies sold as psychedelics or nootropics should be aware that these products might contain undisclosed and potentially harmful substances. Clinicians should be aware that adults who consume these gummies can experience signs and symptoms that include hallucinations, altered mental status, tachycardia, and gastrointestinal upset. Mushroom gummy intoxication might appear similar to cannabis intoxication and can be included in the differential diagnosis of pediatric patients with unexplained somnolence or altered mental status (*5*). Urine drug screens commonly used in health care facilities do not usually detect the substances identified in these gummies^¶¶^; additional testing could be considered based on discussions with a poison control center or local health authorities. 

Persons who purchase products advertised as psychedelic or nootropic mushroom gummies should be aware that package labels might not accurately represent the contents and that these products could contain substances that might produce unexpected and potentially toxic effects. Health care providers should counsel patients and caregivers that mushroom-containing edible products marketed with claims of health benefits might contain undisclosed ingredients and have been linked to illness requiring hospital care. Persons who experience symptoms after consuming these products should seek immediate medical attention.

## Footnotes

* Schedule I substances are defined as drugs with no currently accepted medical use and a high potential for abuse. https://www.dea.gov/drug-information/drug-scheduling

† A naturally occurring psychoactive component found in *A. muscaria* and related mushrooms that displays sedative/hypnotic, depressant, and hallucinogenic activity. The compound works as a gamma-aminobutyric acid type A agonist. https://pubchem.ncbi.nlm.nih.gov/compound/Muscimol

§ A naturally occurring psychoactive compound found in *A. muscaria* and *Amanita pantherine* mushrooms that works as a glutamate agonist. Signs and symptoms of intoxication include agitation, euphoria, and seizures. https://pubchem.ncbi.nlm.nih.gov/compound/ibotenic%20acid

¶ A naturally occurring component of *Inocybe* and *Clitocybe* mushrooms. Found in trace amounts in *A. muscaria*. Signs and symptoms of intoxication include cholinergic symptoms such as headache, nausea, vomiting, and miosis. Effects can be reversed with anticholinergic drugs, such as atropine. https://pubchem.ncbi.nlm.nih.gov/compound/muscarine

** High-resolution mass spectrometry allows for identification of unknown compounds. The technique used for analysis in this report matched various parameters to those in an established library to identify compounds that were then confirmed by comparing the samples with standard materials. This qualitative technique is useful to identify compounds present in a sample. The compounds must be included in the library and be present in the sample at concentrations that allow for accurate identification to be positive. The limit of detection for every compound in the library was not determined. Negative results do not necessarily mean the compound is not present, only that it is unable to be detected.

††
https://nida.nih.gov/research-topics/drug-testing#detect

§§
https://poisoncenters.org/

¶¶
https://emergency.cdc.gov/han/2024/han00509.asp
